# The Influence of Emotion on Fairness-Related Decision Making: A Critical Review of Theories and Evidence

**DOI:** 10.3389/fpsyg.2017.01592

**Published:** 2017-09-19

**Authors:** Ya Zheng, Zhong Yang, Chunlan Jin, Yue Qi, Xun Liu

**Affiliations:** ^1^Department of Psychology, Dalian Medical University Dalian, China; ^2^CAS Key Laboratory of Behavioral Science, Institute of Psychology, Chinese Academy of Sciences Beijing, China; ^3^Department of Psychology, University of Chinese Academy of Sciences Beijing, China; ^4^School of Foreign Languages, East China University of Science and Technology Shanghai, China

**Keywords:** emotion, emotion regulation, fairness-related decision making, fairness theory, neural mechanisms

## Abstract

Fairness-related decision making is an important issue in the field of decision making. Traditional theories emphasize the roles of inequity aversion and reciprocity, whereas recent research increasingly shows that emotion plays a critical role in this type of decision making. In this review, we summarize the influences of three types of emotions (i.e., the integral emotion experienced at the time of decision making, the incidental emotion aroused by a task-unrelated dispositional or situational source, and the interaction of emotion and cognition) on fairness-related decision making. Specifically, we first introduce three dominant theories that describe how emotion may influence fairness-related decision making (i.e., the wounded pride/spite model, affect infusion model, and dual-process model). Next, we collect behavioral and neural evidence for and against these theories. Finally, we propose that future research on fairness-related decision making should focus on inducing incidental social emotion, avoiding irrelevant emotion when regulating, exploring the individual differences in emotional dispositions, and strengthening the ecological validity of the paradigm.

## Introduction

Researchers of decision-making typically regard emotion as impulsive and irrational and neglect its role in decision making (Kahneman and Tversky, 1979; Von Neumann and Morgenstern, 2007). In “normative decision theory,” economic decision making is based on “cold” mathematical calculation, and decision makers are idealized as perfect “rational machines.” However, studies increasingly show that emotion is one of the most important factors in the irrational decision-making process (Hastie, 2001; Sanfey et al., 2006). For example, emotion may guide people’s decision making under conditions of risk and uncertainty and with regard to intertemporal choices, social decisions, and moral decision making (Loewenstein and Lerner, 2003; Rilling and Sanfey, 2011).

Fairness-related decision making is an important issue in the field of psychological decision making (Güth and Kocher, 2014). Experiments on fairness-related decision making have usually been conducted using the classic “Ultimatum Game” (UG) paradigm (Güth et al., 1982). An increasing number of UG studies have revealed that responders tended to sacrifice their own payoffs to decline an unfair offer, especially when they receive an offer that is less than 20% of the total (Güth et al., 1982; Thaler, 1988; Camerer and Thaler, 1995). These irrational rejection behaviors cannot be captured by the economic rationality of utility, in which the responder should accept all offers since receiving at least some money is always preferable to receiving no money.

Some theories, such as “inequity aversion” theory (Fehr and Schmidt, 1999; Bolton and Ockenfels, 2000) and “reciprocity equilibrium” theory (Rabin, 1993; Falk and Fischbacher, 2006), have attempted to explain irrational behaviors in fairness-related decision making. “Inequity aversion” means that people prefer equitable outcomes: they are willing to forego a material payoff to work toward more equitable outcomes (Fehr and Schmidt, 1999; Bolton and Ockenfels, 2000). However, it is difficult to explain why unfair offers from computer partners were accepted at higher rates than human partners if people were pursuing only fairness in terms of their own material payoff relative to the payoff of others (Blount, 1995; Knoch et al., 2006). According to “reciprocity equilibrium” theory, the rejection in the UG with human partners is social punishment to promote fair offers in subsequent bargaining, establish a good reputation, or enforce fairness norms (Rabin, 1993; Falk and Fischbacher, 2006). Thus, people will reject unfair offers from human partners, but accept unfair offers from computer partners to maximize personal gains. One study found that players would reject unfair offers when rejection reduced only their own earning to 0, and even when they cannot communicate their anger to the proposers through rejection (Yamagishi et al., 2009). The rejection of unfair offers that increase inequity and fail to punish proposers cannot be explained by the “inequity aversion” and “reciprocity equilibrium” theories. Such studies have increased awareness of the fact that emotion may be an important reason for irrational behaviors in fairness-related decision making (Sanfey et al., 2003; Ferguson et al., 2014). They propose that rejection is used to express the negative emotions such as anger or disgust aroused by unfair offers (Xiao and Houser, 2005). Although the two classical theories do not deny the existence of emotion, they nevertheless do not clearly explain the role of emotion and its mechanism. A new perspective on emotion is required to explain behavior in fairness-related decision making. Many studies have explored the influence of emotion on fairness-related decision making using behavioral, electrophysiological and neuroimaging approaches and supported these theories.

The influence of emotion on decision making concerns integral emotions (i.e., task-driven) and incidental emotions (i.e., task-unrelated) (Loewenstein and Lerner, 2003). The Wounded Pride/Spite Model suggests that integral emotion, such as negative emotions provoked by unfair offers, prompt rejections (Straub and Murnighan, 1995; Pillutla and Murnighan, 1996). However, this model only focuses on the influence of emotional response aroused by fairness-related decision making; it does not consider the influence of emotion aroused by dispositional or situational sources objectively unrelated to the task. To address this gap, the Affect Infusion Model investigated how incidental emotion (emotion aroused by emotional videos or images) influence fairness-related decision making (Forgas et al., 2003; Bless et al., 2006). These two models emphasized the role of emotion in fairness-related decision making, but ignored the regulation of emotion by cognition in modulating behavior. The Dual-Process System claims that the rational system and the emotional system are dual subsystems in fairness-related decision making, with the former prompting an adaptive response to different situations by regulating the latter (Loewenstein and O’Donoghue, 2004; Sanfey and Chang, 2008; Feng et al., 2015). This review summarizes these models of the impact of emotions on fairness-related decision making and the corresponding behavioral and neural evidence.

## Wounded Pride/Spite Model and its Evidence

### Wounded Pride/Spite Model

The Wounded Pride/Spite Model proposes that the integral emotion aroused by a task itself may change fairness-related decision making. The model claims that if responders perceive that offers are unfair, feelings of wounded pride and anger may be aroused (Straub and Murnighan, 1995; Pillutla and Murnighan, 1996). When direct channels for expressing emotions are either impossible or undesirable, individuals are willing to incur the costs of rejection to retaliate against perceived unfairness (Gross and Levenson, 1993; Gross, 1999). Even when the responder has no way to punish the proposers, the responder still wants to reject the unfair offer (Yamagishi et al., 2009), suggesting that rejection may be not only a strategy to enlarge future potential payoffs but also an effective means of emotional release. However, if responders can convey their feelings of unfairness to proposers, the acceptance rates (ARs) of unfair offers could be increased substantially (Xiao and Houser, 2005).

### Evidence from Integral Emotion

According to a large number of recent studies, the integral negative emotions aroused by unfair offers can increase the punishment for violating fairness norms.

First, previous studies found that fairness-related decision making can evoke strong emotions, demonstrating the existence of integral emotion in fairness-related decision making. From the responders’ self-reports, the researchers found that when responders received an unfair offer, their negative affective responses, such as anger, contempt, irritation, envy and sadness, increased, whereas positive affective responses, such as pleasure and happiness, decreased (Pillutla and Murnighan, 1996; Bosman et al., 2001; Xiao and Houser, 2005; Osumi and Ohira, 2009; Voegele et al., 2010; Hewig et al., 2011; Bediou and Scherer, 2014; Gilam et al., 2015). Researchers used the UG to examine the affective correlates of decision making and found that the decision to reject is positively related to more negative emotional reactions, increased autonomic nervous system and skin conductance activity (van’t Wout et al., 2006; Hewig et al., 2011), and decelerated heart rate (Osumi and Ohira, 2009; Dunn et al., 2012). Furthermore, similar facial motor activities were evoked by unfair treatment, unpleasant tastes, and photographs of contaminants, suggesting that unfairness elicits the same disgust as bad tastes and disease vectors (Chapman et al., 2009).

Second, the affective response to unfairness offers is one possible reason for rejection in fairness-related decision making. Psychophysiological studies have shown that increased ARs of offers correlate with greater resting heart rate variability (Osumi and Ohira, 2009; Dunn et al., 2012). EEG studies found that feedback-related negativity (FRN) could predict the likelihood of rejection in the UG and that rejection was associated with negative emotion (van’t Wout et al., 2006; Hewig et al., 2011). By using the dipole localization method, EEG studies showed that unfair offers could arouse the activation of the insula, which is associated with negative emotion, and the anterior cingulate cortex (ACC), which is associated with conflict monitoring (Guclu et al., 2012). Neuroimaging studies also showed a negative correlation between the activation of the insula specifically involved in aversive emotion and the ARs of unfair offers (Sanfey et al., 2003; Takagishi et al., 2009). The above findings indicate that negative emotion aroused by perceptions of unfairness play an important role in rejection behaviors, supporting the Wounded Pride/Spite Model.

Although the Wounded Pride/Spite Model proposes that negative emotion in fairness-related decision making is an important factor in the rejection of an unfair offer (van’t Wout et al., 2006; Hewig et al., 2011) and can explain many behaviors in fairness-related decision making (Harle and Sanfey, 2007; Grecucci et al., 2013b), this model is only concerned with the responders’ emotional reaction that is aroused by fairness-related decision making. It ignores the impact of the responders’ emotional state and other contextual factors.

## Affect Infusion Model and Evidence

### Affect Infusion Model

The Affect Infusion Model proposes that incidental emotion aroused by task-unrelated sources can significantly influence fairness-related decision making by priming mood-congruent concepts and dispositions (Forgas et al., 1990; Forgas, 2002). For instance, in fairness-related decision making, people must integrate negative (unfair social signals) and positive (financial benefits) information. Positive incidental emotion makes responders more concerned about their own benefits, thus increasing ARs. By contrast, negative incidental emotion makes responders more concerned about unfair offers, thus decreasing ARs (Harle et al., 2012). That is, acceptance or rejection decisions represent the internal rewards and external fairness principles in fairness-related decision making. Positive emotion can enhance cooperation by recruiting a more assimilative, internally focused processing style that promotes selfishness (Forgas et al., 1990). Negative emotion is an alert signal that requires accommodative processing and increases monitoring of the external environment to process potential threats and hazardous stimulation, increasing concern with social norms (Forgas et al., 2003; Bless et al., 2006). For example, sadness provokes pessimistic framing and increases the processing of threatening information, making responders more concerned about the negative consequences of unfairness and the punishment of those who violate the fairness norm (Harle and Sanfey, 2007).

### Evidence of Incidental Emotion

To explore the influence of incidental emotion, many studies have manipulated the affective state by evoking different valences and arousal levels with images and videos. The results showed that participants in a negative emotional state will reject a greater number of unfair offers (Moretti and Di Pellegrino, 2010; Fabiansson and Denson, 2012; Harle et al., 2012; Liu et al., 2016; Riepl et al., 2016), whereas a positive emotional state may reduce or exert no influence on ARs (Harle and Sanfey, 2007; Andrade and Ariely, 2009; Forgas and Tan, 2013a,b; Liu et al., 2016).

Behavioral studies found that on the one hand, when the participants were responders, compared with a neutral group, sad participants reported more negative emotions, such as anger and disgust, when faced with unfair offers and subsequently made more rejections. However, participants who were induced to experience happy emotions accepted more unfair offers (Riepl et al., 2016), with no discernible impact on their decisions (Harle and Sanfey, 2007; Forgas and Tan, 2013a,b; Liu et al., 2016). On the other hand, when the participants were proposers, inducing amusement (compared with sadness) made them more selfish; they also allocated a greater number of points to themselves and had shorter response times (Forgas and Tan, 2013a,b). Neuroimaging studies indicate that incidental sad emotions are regulated by the three main brain regions for emotions, namely, the insula, ACC and striatum. First, compared with participant responses under neutral conditions, the ARs of unfair offers were associated with higher bilateral insula activations in participants who were sad. Insula is typically associated with negative emotions (Paulus et al., 2003; Knutson et al., 2007), suggesting that this region may indicate an aversive response, which may reduce ARs (Harle et al., 2012). Consequently, some researchers have speculated that insula activation can predict the influence of sadness on decision making (Sanfey et al., 2003). Increasing evidence suggests the important role of the anterior insula (AI) in detecting norm violations (Civai et al., 2012; Xiang et al., 2013). Researchers speculated that a sad participant with increased AI activity may experience high sensitivity to norm violation. Thus, sad incidental emotion could activate the insula involved in negative emotion (or detection of norm violation) and bias behavior accordingly. Second, receiving unfair offers in a sad vs. neutral mood resulted in greater activation in the ACC linked to error and decision conflict monitoring, suggesting that sad individuals may experience an enhanced perception of social norm violation (Harle et al., 2012). Furthermore, a moderating effect of mood was found in the left ventral striatum, which is associated with reward processing. Individuals who experienced a neutral mood showed stronger activation for fair offers relative to unfair offers, while individuals who were sad did not exhibit such a pattern of activation, implying decreased reward responsiveness to reward stimuli (Harle et al., 2012). Overall, both behavioral and neural studies have shown that negative emotions enhance participants’ negative responses to behaviors that violate fairness norms and reduce reward activation for fair offers, thus decreasing ARs. These studies demonstrate that emotion plays a role in changing participants’ decisions by altering their cognitive processing, supporting the Affect Infusion Model.

However, some researchers have noted that the dimension of emotional motivation, rather than emotional valence, is the key factor that influences fairness decision making. Emotional valence refers to the intrinsic attractiveness (positive valence) or averseness (negative valence) of an event, an object, or a situation (Frijda, 1986). Emotional motivation refers to the aversive and appetitive apparatuses, which, respectively, promote withdrawal and approach behavior (Schneirla, 1959; Lang et al., 1997). Two emotions with similar valences may have different motivations, and vice versa. For instance, amusement and serenity are positive emotions, whereas anger and disgust are negative emotions. However, amusement and anger are classified as approach-based emotions, whereas serenity and disgust are withdraw-based emotions. Therefore, researchers have suggested that compared with a valence framework, partitioning affective states based on motivational tendency could more accurately explain the changes in ARs in fairness-related decision. The results of a study that explored the influence of positive emotions (amusement and serenity) and negative emotions (anger and disgust) on fairness-related decision making, indicate that emotional valence did not predict ARs. However, the approach-based emotional states (amusement, anger) increased ARs, whereas withdrawal-based emotional states (disgust, serenity) decreased ARs (Harle and Sanfey, 2010). Thus, emotional motivation may help explain fairness-related decision making. Many researchers have explored the emotional influence of fairness-related decision making in terms of approach-based states (anger) and withdrawal-based emotional states (disgust) (Andrade and Ariely, 2009; Moretti and Di Pellegrino, 2010; Liu et al., 2016; Riepl et al., 2016).

Studies have shown that anger influences fairness-related decision making and leads responders to reject more unfair offers. On the one hand, anger functions as a negative emotion after unfair treatment (Pillutla and Murnighan, 1996) and thus decreases the ARs of unfair offers. Prior to a decision, the responders’ anger elicited by watching the video clip made them reject more unfair offers compared with responders who watched a pleasant video clip (Andrade and Ariely, 2009; Riepl et al., 2016). When manipulating the facial expressions of the proposers, the same results were found: responders facing angry proposers provided the most rejections, whereas the least rejections were from those who faced pleasant proposers (Mussel et al., 2013; Liu et al., 2016). When the responder’s anger was provoked by the controlled proposer’s negative appraisal of the responder’s speech, decreased ARs resulted (Fabiansson and Denson, 2012). To the best of our knowledge, only one study used an EEG and explored the neural mechanism of the influence of incident emotion on fairness-related decision making. That study induced anger, fear and happiness via short movie clips. The results showed that responders with high trait negative affect in aversive mood states had increased FRN amplitudes when they were in an angry mood but not when they experienced fear or happiness (Riepl et al., 2016). On the other hand, whether the proposer or the responder is the angry party leads to different perceptions of fairness and judgments of the proposer’s offer. If the proposers are angry, more unfair offers are given. For example, if the proposer’s anger is aroused by the responder, the proposer is more likely to split unfair offers (Fabiansson and Denson, 2012). In contrast, if the responder feels angry, more fair offers are given. For example, proposers will make more fair offers when they know that the responders watched an angry video clip in contrast with the knowledge that the responders watched a happy clip (Andrade and Ho, 2007). The above results may relate to the proposers’ attribution of anger. Anger is a kind of high-arousal and approach-based negative emotion (Berkowitz and Harmon-Jones, 2004; Carver and Harmon-Jones, 2009), and it may cause antisocial behaviors related to revenge (Carnevale and Isen, 1986; Pillutla and Murnighan, 1996; Allred et al., 1997). Therefore, when the responder is the one to irritate the proposer, the proposer proposes more unfair offers in return. Second, anger may make people tougher and more dominant (Knutson, 1996; Tiedens, 2001). People know that angry people are impulsive and act irrationally (Bacharach and Lawler, 1981), so they may make more fair offers to reduce the possibility of being rejected instead of irritating the responder to maximize the profits in bargaining when they play as proposers (Andrade and Ho, 2007; Andrade and Ariely, 2009).

In addition, disgust aroused prior to a decision can increase the responder’s punishment for unfair offers, whereas the idea of misattributing the disgust induced by the unfair offer to incidental disgust will reduce the responder’s punishment. When responders have viewed emotional pictures or faces to arouse aversion prior to a decision, lower ARs to unfair offers are caused by the disgust (Moretti and Di Pellegrino, 2010; Liu et al., 2016). In a comparison of the influence of disgust and sadness on fairness decisions, disgust caused obviously lower ARs (Moretti and Di Pellegrino, 2010). However, another study using disgusting smells showed that participants misattributed the disgust induced by an unfair offer to the disgusting smell, which led to higher ARs (Bonini et al., 2011). These results indicate that the arousal of disgust prompts people’s maintenance of social norms because disgust is a type of withdrawal-based emotion (Harle and Sanfey, 2010) and may be extended to moral and social violations (Rozin et al., 2000). As an indicator of the judgment of others’ behavior as either right or wrong, feelings of disgust can function better than sadness as moral intuition (Haidt, 2001) to decrease the ARs of unfair offers. To an extent, disgust aroused prior to a task overlapped with disgust in the distribution, whereas the attribution of the latter to the former resulted in a subtraction of the emotion.

From the above, we may conclude that the valences of anger and aversion are the same; however, due to the different induction manipulations and attributions, they may have different impacts on fairness-related decision making. Consequently, the Affect Infusion Model takes the motivational direction of emotion as an important factor to interpret the emotional process of fairness-related decision making within a wider range.

## Dual-Process Systems and the Empirical Study

### Dual-Process Systems

The above two models focused on the function of emotional arousal and appraisal in fairness-related decision making but ignored the regulation of emotion by cognition to change decision making. The Dual-process System claims that there are dual subsystems in fairness-related decision making: one is automatic, with an immediate response and an emotional system with no cognitive effort, whereas the other is controlled and comparatively slow, with a rational system of cognitive effort. The emotional system represents the intuitive response; however, after learning and calculation, the rational system requires an adaptive response to different situations by regulating the emotional system (Loewenstein and O’Donoghue, 2004; Sanfey and Chang, 2008; Feng et al., 2015). Fairness-related decision making is influenced by systematically and effectively regulating responders’ fairness perceptions via rational cognitive control (Rilling and Sanfey, 2011). For example, the model suggests that all types of emotional regulation strategies can change fairness-related decision making through the interaction of cognition and emotion.

### The Empirical Study of Dual-Process Systems

Researchers have employed different emotion regulation strategies and compared their effectiveness. The results support the influence of emotion regulation on fairness-related decision making.

First, responders may spontaneously regulate the negative emotions induced by unfair offers in the UG. After decision making, responders are requested to report their own opinions on the offer and to write down the shift of their decisions as follows: “At the very beginning, I thought of…, then I considered.…” Some responders may remain angry, reject the unfair offer and refuse to report, whereas others may spontaneously employ cognitive reappraisal to reduce their own negative emotions and then accept more unfair offers (Voegele et al., 2010; Gilam et al., 2015). In physiological arousal, responders who employed reappraisal showed higher vagal activation and attenuated heart rate deceleration after accepting unfair offers (Voegele et al., 2010). Neuroimaging studies have revealed that increased ARs of unfair offers are associated with increased activity in the ventrolateral prefrontal cortex (vlPFC), a region involved in emotion regulation, and decreased activity in the AI, which is linked to negative affect (Tabibnia et al., 2008). Individuals with high monetary gains showed increased ventromedial prefrontal cortex (vmPFC) activity but also decreased AI activity (Tabibnia et al., 2008; Gilam et al., 2015). Furthermore, patients with vmPFC damage had lower ARs than control groups (Koenigs and Tranel, 2007). The studies suggested that brain areas associated with emotion regulation, such as vlPFC and vmPFC, may be engaged to diminish the aversion-related AI’s response (Tabibnia et al., 2008; Gilam et al., 2015) and increase the ARs of unfair offers.

Second, multiple emotion regulation strategies can change decisions by regulating emotions. Researchers have employed two strategies for emotion regulation in fairness-related decision making: reappraisal and expressive suppression. The results showed that although the two strategies could reduce the negative emotions of responders to unfair offers, though compared with expressive suppression, the reappraisal strategy was more effective in changing responders’ emotions and making them accept more unfair offers (Kirk et al., 2006; van’t Wout et al., 2010; Fabiansson and Denson, 2012). In addition, reappraisal strategies may continue to reduce participants’ negative emotions and make them propose more fair offers during a second interaction with partners who treated them unfairly in a previous interaction, whereas the expressive suppression strategy may reduce participants’ previous negative emotions with no effect of ridding themselves of negative treatment, resulting in the proposal of unfair offers (van’t Wout et al., 2010; Fabiansson and Denson, 2012). The results showed that to change emotions and behaviors using an emotion regulation strategy and to avoid previous negative impact, the reappraisal strategy is considerably more effective than expressive suppression and can extend beyond a single encounter to influence future interaction. Grecucci furthered the study of reappraisal strategies by discussing up- and down-regulation (Grecucci et al., 2013b). The former refers to the interpretation of intentions and behaviors of unfair offers as more negative (i.e., the player is a selfish person and wants to keep all the monetary gains), whereas the latter refers to these as less negative (i.e., the proposers’ debt problems leading them to gain more). The results showed that responders with an up-regulation strategy rejected more unfair offers in contrast with down-regulation, demonstrating that reappraisal strategies may change the way responders understand others’ intentions and affect their emotional reaction, resulting in changed decisions. Overall, the reappraisal strategy can modulate the impact of emotional stimuli, contributing to our decisions flexibly (Grecucci et al., 2013b). Neuroimaging studies revealed that the dorsolateral prefrontal cortex (DLPFC) and bilateral ACC play vital roles in the reappraisal process. The DLPFC is associated with cognitive control and inhibition (Miller and Cohen, 2001) as the basis of the generation and maintenance of reappraisal strategies (Ochsner et al., 2002; Ochsner and Gross, 2005). Additionally, Buckholtz et al. (2008, 2015), Buckholtz and Marois (2012) proposed the integrative model of DLPFC function, which suggested the role of DLPFC in the representational integration of the distinct information streams used to make punishment decisions. When applying cognition reappraisal in fairness-related decision making, the evaluation of fairness and the information concerning harm and blame changed. Therefore, the DLPFC activated to integrate the information from emotional response, regulation strategy, fairness evaluation and other sources to make punishment decisions. Furthermore, the ACC monitors and evaluates conflicting responses or motives (Yeung and Sanfey, 2004; Ochsner and Gross, 2005).

In addition to reappraisal and expressive suppression, expected emotion is an effective way of regulating fairness-related decision making. With regard to changing a decision, some studies have investigated the regulation of individuals’ expected emotion induced by the decision outcome. In the decision stage, responders will attempt to predict the probabilities of different outcomes and the emotional consequences associated with alternative actions. To minimize negative emotion and maximize positive emotion, responders will adjust their decisions (Loewenstein and Lerner, 2003; Rick and Loewenstein, 2007). If they predict they will be proud of their fair offers, more fair offers will be given, whereas if they predict that they will feel regretful, less fair offers will be chosen. The expected emotion helps them to anticipate future outcomes and modify their behaviors to evoke desirable emotions and avoid undesirable results. When an individual can expect a positive outcome, it is likely that a current offer will be supported. In contrast, an expected negative outcome will lead to modification of the current activities (Baumeister et al., 2007). Some researchers have manipulated the expected emotion using the autobiographical recall task and found that anticipated pride about fair behavior increased levels of fairness, whereas anticipated pride about unfair behavior decreased levels of fairness. Similarly, anticipated regret about fair behavior reduced levels of fairness, whereas anticipated regret about unfairness increased levels of fairness (van der Schalk et al., 2012). If the proposers were required to observe pride or regret after making fair or unfair offers in the UG, they made fewer fair offers if they had seen the responder’s regret about a fair offer, whereas they made more fair offers if they had seen the responder’s regret about unfair offers (van der Schalk et al., 2014). The results showed that past emotional experience make people reflect on and modify the outcome of their behavior because they pursue not only maximized benefits but also positive emotional experiences (Mellers et al., 1999; Loewenstein and Lerner, 2003). Other studies on regulating strategies of delay or distraction revealed that the delay of a decision did not change the emotional experience or behavior (Bosman et al., 2001), whereas distraction only decreased anger but did not change fairness-related or other decisions when anger was induced again by the same stimulus (Gross and Levenson, 1993; Gross, 1999; Xiao and Houser, 2005; Fabiansson and Denson, 2012).

Neural mechanism studies on the emotion regulation of fairness-related decision making have supported Dual-process Systems. The interaction of the automatic processing emotional system and the controlled cognitive system affects people’s behavior. The emotional system includes the insula, which is associated with aversion to violating norms (Sanfey et al., 2003; Guo et al., 2013); the amygdala, which is associated with negative emotions (Haruno and Frith, 2010; Haruno et al., 2014); and the vmPFC, which is associated with encoding subjective values of perceived offers and emotion regulation (Tabibnia et al., 2008; Baumgartner et al., 2011; Gilam et al., 2015). In addition, the controlled cognitive system involves the dorsal ACC, which regulates the conflict of norm enforcement and self-interest and DLPFC (Knoch et al., 2006, 2008; Baumgartner et al., 2011) related to executive control.

Dual-process Systems focus on the function of emotions and involve the interaction of emotion and cognition for fairness-related decision making. This model has been supported by many behavioral and neuroimaging studies (Sanfey et al., 2003; Baumgartner et al., 2011). This model also proposes strategies for regulating emotion that provide a new way of changing fairness-related decision making (Knoch et al., 2006, 2008). However, current evidence is limited to the regulation of negative emotion induced by an offer (Grecucci et al., 2013a,b). Little is known about the regulation of incidental emotion in fairness-related decision making.

## A Schematic Illustration of the Influence of Emotion on Fairness-Related Decision Making

**FIGURE 1:**
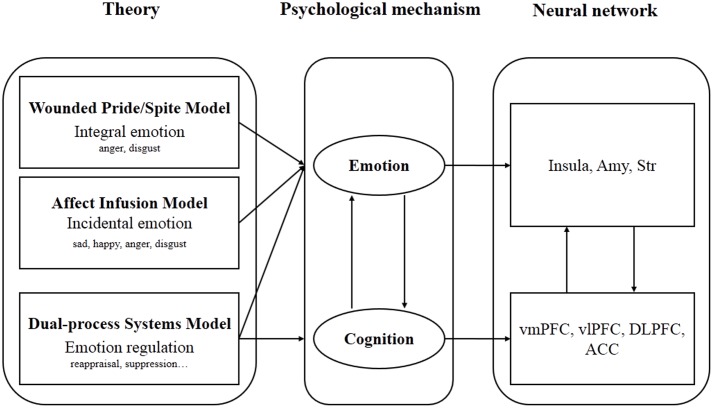
A schematic illustration of the influence of emotion on fairness-related decision making. The Wounded Pride/Spite Model and the Affect Infusion Model describe the influence of emotion, whereas the Dual-Process Systems Model proposes that cognition could regulate emotion to regulate fairness-related decision making. The related brain regions include the insula, amygdala (Amy), striatum (Str), ventrolateral prefrontal cortex (vlPFC), ventromedial prefrontal cortex (vmPFC), dorsolateral prefrontal cortex (DLPFC), and anterior cingulate cortex (ACC).

In complex social environments, both the emotion and cognition systems are involved in processing the fairness perception of resource distribution (see **Figure 1**). The Wounded Pride/Spite Model and the Affect Infusion Model describe the influence of integral emotion aroused by task and incidental emotion aroused by task-unrelated resources, respectively. For instance, compared with fair offers, unfair offers have been associated with greater activation of the insula, which is involved in aversion emotion (Sanfey et al., 2003; Takagishi et al., 2009; ?), whereas fair offers have been linked to the activation of reward regions, such as the ventral striatum (Tabibnia et al., 2008). Additionally, individuals in sad or angry moods showed an enhanced perception of unfairness, with a greater activation of the insula and amygdala (Harle et al., 2012). The Dual-process Systems perspective proposes that the rational system could regulate emotion to both up- and down-regulate fairness-related decision making. For example, the ACC monitors and evaluates conflicts between norm enforcement and financial benefit (Yeung and Sanfey, 2004; Ochsner and Gross, 2005). The vlPFC and vmPFC associated with emotion regulation could decrease the activation of AI to diminish conflicts (Tabibnia et al., 2008; Gilam et al., 2015). The DLPFC is associated with cognitive control and inhibition (Miller and Cohen, 2001) and influences generation and maintenance reappraisal strategies (Ochsner et al., 2002; Ochsner and Gross, 2005). It can integrate the information from emotional response, regulation strategy, fairness evaluation and other sources to make punishment decisions (Buckholtz and Marois, 2012).

## Summary and Prospects

In the history of studies on fairness-related decision making, the hypothesis has changed from viewing responders as completely rational with no influence from emotion to regarding both emotion and cognition as important factors in Dual-process Systems. Many studies have revealed that emotion plays an important role in fairness-related decision making. Based on the review of the theoretical and empirical studies, we conclude that the future research scope of the influence of emotion in fairness-related decision making can be furthered in the following ways.

First, recent studies that have induced incidental emotions are limited to several basic emotions, such as happiness, sadness, anger or disgust. However, as a social animal, humans have complicated, delicate and vast social structures and interpersonal relations. Among these, social emotions are one of the important motivations for human behavior. Since fairness is one of the basic norms in human society, it is influenced by many social emotions (Takahashi et al., 2009). As a result, future research should explore the impact of social emotions, including both positive social emotions (empathy, gratitude) and negative social emotions (envy, indignation), on fairness-related decision making.

Second, reappraisal is a common strategy to regulate emotional response, but this strategy involves reinterpreting the meaning of a stimulus. In studies on fairness-related decision making, responders can adopt an up-regulation strategy or a down-regulation strategy. Responders must evaluate the motivations and behaviors of proposers to decrease the anger or disgust caused by unfair offers (Grecucci et al., 2013b). However, reappraisal may induce other emotions, such as empathy from down-regulation (Gross, 2013). Future studies should aim to identify the irrelevant emotions aroused by the regulation strategy that may influence fairness-related decision making.

Third, some personal traits, such as emotional dispositions (Dunn et al., 2010), social value orientation (Karagonlar and Kuhlman, 2013; Haruno et al., 2014), and personality characteristics (Spitzer et al., 2007; Osumi et al., 2012), may influence personal emotional response and regulation, thus affecting fairness-related decision making. For this reason, we suggest that future studies should explore the possible interaction of personality traits, emotion and unfair offers.

Finally, the standard UG paradigm has been widely used in studies on the influence of emotions on fairness-related decision making. Some complex, modified versions of the UG may complicate the context of fairness-related decision making, but may nevertheless be accurate models of real-world situations. For instance, we can put fairness-related decision making in the more complex background of social comparison (Wu et al., 2011; Alexopoulos et al., 2012; McDonald et al., 2013), the loss context (Buchan et al., 2005; Zhou and Wu, 2011; Guo et al., 2013), or making responders perceive the intentions of the proposer (Radke et al., 2012; Ma et al., 2015). As a result, future studies on the influence of emotions on fairness-related decision making should consider ecological validity to make the studies more realistic.

## Author Contributions

Conceived and designed the study: ZY, YZ, and XL. Literature search and synthesis: CJ and YQ. Wrote the paper: ZY and YZ.

## Conflict of Interest Statement

The authors declare that the research was conducted in the absence of any commercial or financial relationships that could be construed as a potential conflict of interest.
